# Efectos del tratamiento ortopédico en la articulación temporomandibular en pacientes clase III con mordida cruzada anterior: una revisión de literatura

**DOI:** 10.21142/2523-2754-1103-2023-166

**Published:** 2023-09-26

**Authors:** Karen Moscoso Sivirichi, Roxana Gissella Gutiérrez Tapia

**Affiliations:** 1 División de Ortodoncia, Universidad Científica del Sur. Lima, Perú. karenmsivirichi@gmail.com, dentalgissella@yahoo.es Universidad Científica del Sur División de Ortodoncia Universidad Científica del Sur Lima Peru karenmsivirichi@gmail.com dentalgissella@yahoo.es

**Keywords:** ortopedia, articulación temporomandibular, trastornos de la articulación temporomandibular, maloclusión, maloclusión de Angle clase III, orthopedics, temporomandibular joint, temporomandibular joint disorders, malocclusion, Angle Class III malocclusion

## Abstract

**Objetivo:**

El estudio tuvo como objetivo evaluar los efectos del tratamiento ortopédico en la articulación temporomandibular (ATM) en pacientes clase III con mordida cruzada anterior.

**Materiales y métodos:**

Cuatro bases de datos fueron revisadas hasta diciembre de 2022 (Scopus, PubMed, Embase y ScienceDirect). Dos revisores independientes seleccionaron estudios que reportaran cambios en el ATM y presencia de disfunción de la ATM en pacientes clase III con mordida cruzada anterior que recibieron tratamiento ortopédico. La calidad metodológica de los estudios fue analizada usando PRISMA *checklist*.

**Resultados:**

Se seleccionaron doce artículos para el estudio después de una búsqueda detallada de acuerdo con los criterios de selección. Después del tratamiento, los estudios demostraron una baja prevalencia de dolor en la ATM tras el uso de la máscara facial de Delaire, de la mentonera, del *jasper jumper* modificado y el *twin block* reverso fijo. Después del tratamiento con *twin block* reverso, se evidenció un aumento de volumen condilar y de los espacios articulares (p < 0,001), seguido por una disminución significativa de los espacios articulares posterosuperiores (p < 0,001).

**Conclusiones:**

No se ha encontrado suficiente evidencia que determine presencia de DTM o cambios en la ATM después del uso de aparatos ortopédicos para corrección de mordida cruzada anterior.

## Abstract (en)

**Objective:**

The study aimed to evaluate the effects of orthopedic treatment on the temporomandibular joint (TMJ) in class III patients with anterior crossbite.

**Materials and Method:**

Four databases were reviewed until December 2022 (Scopus, PubMed, Embase, and Science Direct). Two independent reviewers selected studies reporting TMJ changes and the presence of TMJ dysfunction in Class III patients with anterior crossbite who received orthopedic treatment. The methodological quality of the studies was analyzed using the PRISMA checklist.

**Results:**

Twelve articles were selected for the study after a detailed search according to the selection criteria. Post-treatment studies demonstrated a low prevalence of TMJ pain after using the Delaire face mask, chin rest, modified Jasper Jumper, and fixed reverse Twin block. After treatment with reverse Twin block, an increase in condylar volume and joint spaces (p<0.001) was observed, followed by a significant decrease in posterosuperior joint spaces (p<0.001).

**Conclusions:**

Not enough evidence has been found to determine the presence of TMD or TMJ changes after using orthopedic devices for anterior crossbite correction.

## INTRODUCCIÓN

La mordida cruzada anterior (MCA) es considerada un signo característico de la maloclusión clase III, que se produce por un retrognatismo maxilar o prognatismo mandibular, y se caracteriza por presentar un *overjet* negativo en uno o más dientes anterosuperiores e inferiores ^1^^,^^2^. Su etiología es multifactorial, es decir, puede ser genético, por la alteración en el crecimiento de los maxilares, la posición de la lengua y la presión muscular del labio y lengua ^3^^).^

Esta afección altera la estética facial y la función masticatoria, inclusive se ha demostrado que cuando no es tratada a tiempo puede ocasionar desorden de la articulación temporomandibular (ATM) y alteración muscular, así como una asociación con la desviación mandibular y la mordida cruzada posterior unilateral ^4^^-^^7^. Existen diversos aparatos para la corrección de la MCA, entre ellos los aparatos de protracción maxilar ^8^^,^^9^, la mentonera ^10^^,^^11^ y los aparatos funcionales, como el *jasper jumper* modificado, Frankel III, Bimler C, los bloques gemelos reversos y Yanagisawa C3 (YC3).^3^^,^^12^.

Algunos estudios demostraron cambios en el ATM después del tratamiento con aparatos ortopédicos, entre ellos los aparatos de protracción maxilar y aparatos funcionales, como el *jasper jumper* modificado, los cuales presentaron un cambio en el cóndilo más superior y posterior de la cavidad glenoidea pero no se ha considerado la presencia de disfunción temporomandibular (DTM) por no ser altamente significativo ^13^^-^^18^. Por otra parte, los bloques gemelos reversos que evidenciaron una retrusión de la mandíbula no han demostrado que inducen a una DTM en pacientes adultos sin síntomas ^19^. Otros estudios evidenciaron que la mentonera produciría una luxación del disco del ATM con reducción de este, e inclusive un cambio en la morfología del cóndilo ^17^^,^^18^^,^^20^.

Por ello, este estudio tuvo como propósito identificar los efectos que ocurren en la articulación temporomandibular después de la corrección de la mordida cruzada anterior con aparatos ortopédicos.

## MATERIALES Y MÉTODOS

### Estrategia de búsqueda

Este estudio de revisión de literatura fue recogido de cuatro bases de datos: PubMed, Embase, Scopus y ScienceDirect, publicados hasta octubre de 2022. En esta búsqueda se consideró descriptores MeSH (*Medical Subject Heading*) y palabras claves en los títulos o resúmenes, utilizando operadores booleanos en la búsqueda ("temporomandibular joints" OR TMJ OR TMD) AND (crossbite OR "anterior crossbite" OR "Class III" OR "III malocclusion" OR "mandibular prognathism") AND (orthopedic OR facemask OR protraction OR techniques). Además, se usó el gestor de referencias RefWorks para evitar cualquier información idéntica (Tabla 1).

**Tabla 1:** Estrategia de búsqueda de descriptores de las diferentes bases de datos

PubMed (13/12/2022)
n = 21
"temporomandibular joints"(title/abstract) OR TMJ(title/abstract) OR TMD(title/abstract)) AND (crossbite(title/abstract) OR "anterior crossbite"(title/abstract) OR "Class III"(title/abstract) OR "III malocclusion"(title/abstract) OR "mandibular prognathism"(title/abstract)) AND (orthopedic(title/abstract) OR facemask(title/abstract) OR protraction(title/abstract) OR techniques(title/abstract)
Scopus (13/12/2022)
n = 104
TITLE-ABS-KEY ( ( "temporomandibular joints" OR tmj OR tmd ) AND ( crossbite OR "anterior crossbite" OR "Class III" OR "III malocclusion" OR "mandibular prognathism" ) AND ( orthopedic OR facemask OR "protraction" OR techniques ) ) AND ( LIMIT-TO ( DOCTYPE , "ar" ) ) AND ( LIMIT-TO ( SUBJAREA , "DENT" ) )
Embase (13/12/2022)
n = 14
('temporomandibular joints':ti,ab,kw OR tmj:ti,ab,kw OR tmd:ti,ab,kw) AND ('anterior crossbite':ti,ab,kw OR 'class iii':ti,ab,kw OR 'iii malocclusion':ti,ab,kw) AND (orthopedic:ti,ab,kw OR facemask:ti,ab,kw OR protraction:ti,ab,kw)
ScienceDirect (13/12/2022)
n = 7
("temporomandibular joints" OR TMJ OR TMD) AND ("crossbite" OR "Class III" OR "III malocclusion") AND (orthopedic OR facemask OR protraction)

### Criterios de búsqueda

La búsqueda de literatura se basó en la pregunta PICO: P (pacientes clase III con mordida cruzada anterior), I y C (diferentes aparatos ortopédicos), y O (patologías en la articulación temporomandibular). Los criterios de inclusión fueron investigaciones en donde el grupo de estudio sea de 30 personas como mínimo, investigaciones que estudien la maloclusión clase III con mordida cruzada anterior, estudios que evalúen la patología de ATM antes y después del tratamiento de ortopedia, estudios longitudinales o comparativos, y estudios publicados en inglés. Los criterios de exclusión fueron investigaciones que evaluaron individuos con cirugía ortognática o tratamiento ortoquirúrgico, investigaciones que evaluaron a individuos con malformaciones congénitas y síndromes, revisiones narrativas, revisiones sistemáticas o metaanálisis, estudios descriptivos, otros estudios (como cartas al editor, editoriales, artículos de opinión, reporte de casos), artículos de investigación duplicados y aquellos que no presentaban texto completo.

### Recolección de datos

**Figura 1:**
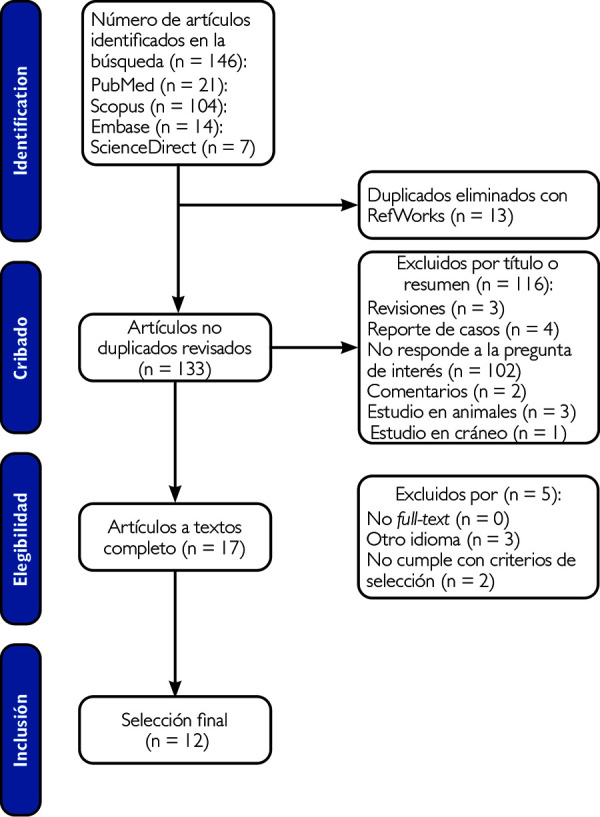
Diagrama de flujo PRISMA de la revisión de la literatura

Los estudios recolectados fueron analizados con base en títulos y resúmenes, según los criterios de selección. Esta revisión fue realizada de forma independiente por una investigadora principal (KMS). En el caso de que el artículo no contara con resumen, se evaluó el texto completo. Las dudas o discrepancias de la selección fueron resueltas por la segunda autora (GRGT). La data de artículos y los motivos de exclusión fueron registrados en archivos. El flujo de trabajo para la selección de artículos siguió los criterios PRISMA (Preferred Reporting Items for Systematic Reviews and Meta-Analyses) ^21^ (Fig. 1).

### Resultados de interés

La investigadora principal (KMS) sintetizó los datos de los estudios seleccionados sobre autores, país, año, diseño de estudio, tamaño de muestra (sexo), rango de edad, características clínicas, tipo de intervención, magnitud y duración de fuerza, periodo de observación, variable respuesta y resultados. La relación final de artículos se recopiló según los resultados de interés (Tabla 2).

**Tabla 2:** Tabla de extracción de información

Id	Autor, país, año	Diseño de estudio	Tamaño de muestra (n, mujeres y hombre)	Rango de edad (años)	Características clínicas	Intervención			Variables respuesta	Resultados
						Tipo de intervención	Magnitud y duración de fuerza (g)	Periodo de observación		
1	Alarcón *et al*., España, 2011	Retrospectivo	90 pacientes (45 varones, 45 mujeres)	T0 GM 8,5 ± 0,5 años y GC 8,6 ± 0,4 años; T1 GM 11,4 ± 0,6 años y GC 11,7 ± 0,5 años	Clase III con MCA, prognatismo mandibular, maxilar normal, caucásico, sin TMJ	50 M (Dentaurum^®^, Ispringen, Alemania), 40 GC	GM: 300 g por lado tracción occipital, 14h/día	M y GC: 3 años	Cefalometría Lateral (Orthotomograph-10, Trophy OPX/105, Trophy Radiologie, Marne la Vallée, Francia; 90kV, 10-15mA) y software tpsDIG (tpsSeries^®^, J. F. Rohlf, Department of Ecology and Evolution, State University of New York at Stony Brook)	Los efectos de M fueron compresión anteroposterior de la distancia relativa entre los cóndilo y proceso coronoides (p < 0,01).
2	Arat *et al*., Turquía, 2003	Prospectivo	124 pacientes (58 varones,66 mujeres)	T0 GT 8,3-14 años, GEIII 12,5-31,1 años y GON 18-21,4 años; T1 GT 9,7-16,8 años	GT Clase III, ANB -1,8°; overjet -1,7 mm; GoGn/SN 30,5° y GEIII ANB -2,8° ± 2,4; overjet -1,8 mm ± 2,4	32 GT, 39 GEIII, 53 GON	GT: 500 g, 14h/día	GT 2-11 años (M = 5 años 6 meses)	Examen funcional (Graber *et al*., 1997; Okeson, 1998): clic, dolor y desviación	Los individuos sintomáticos fueron más en el GON (41,5%) en comparación con el GEIII (23%) (p < 0,05). El dolor en la ATM fue diferente entre GT (37,5%) y GON (54,5%) (p < 0,01). El dolor en la ATM fue mayor en GON (33%) que en GEIII, pero no fue estadísticamente significativo. Los pacientes sintomáticos en el GT (23%) y GEIII (23%) fueron estadísticamente iguales (p < 0,05) La ocurrencia de desviación y clic no difiere entre los grupos (p > 0,05).
3	Deguchi *et al*., Japón, 1998	Prospectivo	86 pacientes (NE)	T1 M 10 años 1 mes	Clase III con MCA	86 M con o sin arco transpalatino	M: 400-500 g tracción occipital, 7-14 h/día	M 6 meses-4 años	Cuestionario de ATM, examinación clínica del ATM	No se encontró signo ni síntoma de DTM en el 67,4% (58 de 86) en T1, el 33% (28 de 86) presentaron uno o más síntomas de DTM (5 varones, 23 mujeres, rango de 10-25 años) en T1. Dolor espontáneo en ATM fue del 3% aprox. en T1; mientras que el clic en la ATM fue del 9%. Los sonidos articulares y alteración en la apertura no fueron observados en T1.
4	Deguchi *et al*., Japón, 1999	Retrospectivo	42 (0 varones, 42 mujeres)	T0 M 9 años 4 meses y GC 9 años 7 meses; T1 M 11años 3 meses y GC 11 años 8 meses	Clase III con MCA	22 M + arco transpalatino, 20 GC	GM: 200-250 g por lado tracción occipital, 7-9 h/día uso nocturno	M 28 ± 7,9 meses, GC 30 ± 12,0 meses	Cefalometría lateral computarizada de coordenadas x-y (Hoya Medical Co., Tokio, Japón)	No hay diferencia significativa que se haya observado en el movimiento horizontal del cóndilo. Condylion se movió menos verticalmente en el M (-0,1 mm por año) en comparación con GC (-0,5 mm por año) (p > 0,05).
5	Hakan *et al*., Turquía, 2010	Prospectivo	34 pacientes (19 varones, 15 mujeres)	T0 MFD 9,03 ± 0,82 años; MFG 9,2 ± 1,1 años	Clase III con MCA no funcional, aparente deficiencia maxilar, sin perdida dentaria, estadio 2 o 3 de maduración vertebral	18 MFD, 16 MFG	MFD y MFG: 300-350g por lado, 14-16 h/dia, 20°-25° vector de fuerza	MFD 8,5 meses; MFG 10 meses	PC en articulador Amtech MG1 (American Technologies, Brasil) antes y después de la terapia de protracción maxilar	Se encontró disminución altamente significativas para ∆X y ∆Z en ambos grupos de T0 a T1 (p < 0,01) de MI a RC; de T0 a T1 hubo diferencias significativas en ∆X del lado derecho e izquierdo de MI a RC y ∆Z del lado izquierdo de la MFD que la MFG, de MI a RC (p = 0,046; 0,044 y 0,007, respectivamente). No hubo diferencias significativas del lado derecho de ∆Z y ∆Y (p = 0,075 y 0,918, respectivamente), evaluados de MI a RC.
6	Khwanda *et al*., Siria, 2022	ECA	40 pacientes (28 varones, 12 mujeres)	T0 9-11 años; T1 TBR 10,12 ± 0,84 años y TBR + TFBM 10,27 ± 0,80 años	Relación molar clase III con MCA, edad 9-11 años, SNA ≤ 0, ausencia de dientes extraídos o faltantes congénitos, sin deformidad en el complejo craneofacial, patrón de crecimiento horizontal y sin antecedentes de trastornos de la articulación temporomandibular	20 TBR, 20 TBR+TFBM	22h/día excepto durante las comidas.	TBR 258,68 ± 16,43 días; TBR + TFBM 193,53 ± 15,34 días	3D CBCT (Pax-i3D Green, Vatech, Seúl, Corea)	En T1 hubo un mayor aumento de la distancia entre espacios articulares en el grupo RTB en comparación con el TBR + PBMT (p < 0,05), un aumento de volumen condilar solo en TBR (p < 0,001), tanto el espacio articular anterior como mediolateral mostraron un aumento estadísticamente significativo en ambos grupos de tratamiento (p < 0,001), así como una disminución significativa de los espacios articulares superior y posterior en ambos grupos (p < 0,001), y hubo mayores cambios en espacios articulares en el grupo RTB (p < 0,001).
7	Kurt *et al*., Turquía, 2010	ECA	46 pacientes (23 hombres, 23 mujeres)	T0 JJM 9,67 ± 0,95 años; MFD 9,55 ± 0,97 años; GC 9.55 ± 0.97 años	Clase III con MCA, retrognatismo maxilar, patrón de crecimiento horizontal (S-N/Go-Me: 30°-32°)	16 JJM, 17 MFD, 13 GC	MFD 400g por lado, JJM 200g por lado, 14h /día ambos grupos	JJM 4,90 ± 0,37 meses; MFD 6,41 ± 0,50 meses; GC 6,00 ± 0,00 meses	Índice RDC/TMD; Cuestionario de dos ejes utilizados para el examen y diagnóstico.	DTM fue observado en cuatro pacientes T0 y T1 en JJM (p > 0,05), DTM fue observado dos pacientes T0 y tres pacientes T1 en MFD (p > 0,05). En el GC, dos pacientes presentaron DTM T0 yT1 (p > 0,05). El diagnóstico en T1 más frecuente fue la artralgia en tres pacientes en JJM y MFD (p > 0,05). En JJM se observaron una disminución del número de ATM dolorosas en T1 en comparación con T0 (p < 0,001).
8	Liu *et al*., China, 2013	Prospectivo	32 pacientes (19 varones, 13 mujeres)	T0 18-28 años	Clase III con MCA de al menos 4 dientes, con o sin dolor, chasquido o cefalea en ATM o DTM, mordida a bis a bis en RC	32 TBRM	NE	14 meses (7-20 meses)	Examinación clínica del ATM	Las observaciones clínicas revelaron que la disfunción de la ATM en la mayoría de los pacientes se alivió significativamente al cabo de 1 mes. Después de 4-5 meses de tratamiento de mantenimiento con la TBR fija, el chasquido articular, el dolor y la cefalea mejoraron o desaparecieron en 18 pacientes. No se observó DTM en pacientes sin síntomas en T0. En un periodo de seguimiento de 3 años, 12 pacientes revelaron OJ y OB normal, sin DTM.
9	Mandall *et al*., Reino Unido, 2012	ECA	63 pacientes (30 varones, 33 mujeres)	T0 GC 9,0 (DE 0,8 años) y MFD 8,7 (DE 0,9 años), T2 MFD 12,1 ± 0,9 años; GC 12,3 ± 0,8 años	Clase III con MCA de 3-4 incisivos, retrognatismo maxilar, caucásicos, 7-9 años, sin signos ni síntomas de ATM	33 MFD (TP Orthodontics®), 30 GC	MFD: 400g por lado, 30º vector de fuerza, 14h/día	MFD y GC: 3 años	Examinación clínica del ATM	Baja prevalencia de signos y síntomas de ATM en ambos puntos de tiempo en el MFD (del 2%, aproximadamente, al 0%). La crepitación fue el signo frecuentemente observado en T2 (11,2% GC y 9,3% MFD).
10	Mandall *et al*., Reino Unido, 2010	ECA	73 pacientes (34 varones, 39 mujeres)	T0 GC 9,0 (DE 0,8 años) y MFD 8,7 (DE 0,9 años). T2 GC 10,3 (DE 0,8 años) y MFD 10,0 (DE 0,9 años)	Clase III con MCA de 3-4 incisivos, retrognatismo maxilar, caucásicos, 7-9 años, sin signos ni síntomas de ATM	35 MFD (TP Orthodontics^®^), 38 GC	GDMF: 400g por lado, 30º vector de fuerza, 14 h/día	MFD y GC: 1 año y 3 meses	Examinación clínica del ATM	Hubo disminución en ambos grupos y tiempos (≤3%) de pacientes con dolor intraarticular, bloqueo y pérdida de movimiento o espasmo masetero/temporal. El dolor lateral en el ATM y espasmo pterigoideo lateral fue ≤ 8,6% en ambos grupos y tiempos. El clic aumentó ligeramente en el MFD de T1 (del 5,7% al 9,1%) y la crepitación aumentó en el GC de T1 (del 5,3% al 15,8%).
11	Mimura *et al*., Japón, 1996	Prospectivo	35 pacientes (12 varones, 23 mujeres)	T0 M 7-13 años y GC 7,9-12 años; T1 M 12,7-18 años y GC 11,8-18,1 años	Clase III leve con MCA, GC: MCA funcional y tamaño mandibular normal	19GM, 16 GC (Arco lingual de Mershon modificado)	NE	M 5 meses-5 años 11meses (M = 2 años 1 mes)	Las artrotomografías sagitales se obtuvieron con un laminógrafo cefalométrico (Sectograph; Quint Co. LTD, Los Ángeles, California)	En M, hubo diferencias significativas en T0 y T1 en el ángulo de la cabeza del cóndilo, altura de cóndilo y espacio anterior del cóndilo (p < 0,05). La altura de la fosa y espacio anterior del cóndilo entre T0 y T1 (p < 0,001). En GC hubo diferencias significativas (p < 0,05) en T0 y T1 en la altura del cóndilo. En T1 hubo diferencias significativas en ángulo de la cabeza del cóndilo, altura de la fosa, ancho de la fosa, espacio anterior del cóndilo y altura del cóndilo en ambos grupos (p < 0,05).
12	Wendl *et al*., Austria, 2017	Retrospectivo	61 pacientes (41 varones, 20 mujeres)	T0 7,8 ± 1,7 años; T1 9,6 ± 2,4 años; T2 15-20 años	Síndrome de Clase III (overjet negativo, Witts < -1mm, ANB negativo, maloclusión clase III)	38 GM, 23 GPM	M 600 g por lado; 24 h/día durante tratamiento y uso nocturno postratamiento. GPM 300 g por lado, 30° vector de fuerza	M y GPM: 6-9años	M: radiografía panorámica, examinación clínica funcional, índice de disfunción de Graz	Tres pacientes mostraron cambios condilares en T1 (p > 0,05). Ninguno de los pacientes reveló ningún signo o síntoma que cumpla con los criterios de una función anómala definida por el índice de disfunción de Graz (p < 0,05).

ECA: ensayo clínico aleatorizado, MCA: mordida cruzada anterior, MFD: grupo Delaire máscara facial, GC: grupo control, MFG: Grupo Grummons máscara facial, PC: posición condilar, ∆X: distancia anteroposterior, ∆Y: distancia mediolateral, ∆Z: distancia superoinferior, MI: máxima intercuspidación, RC: relación céntrica, JJM: grupo *jasper jumper* modificado, RDC/TMD: criterios diagnósticos de investigación para los trastornos temporomandibulares, DTM: desórdenes temporomandibulares, T0: inicio del tratamiento, T1: final del tratamiento, DE: desviación estándar, T2: seguimiento, NE: no especifica, M: grupo mentonera, GT: grupo de tratamiento, GEIII: grupo clase III esqueletal, GON: grupo oclusión normal, TBR: grupo *twin block* reverso, TFBM: terapia fotobiomodulador, TBRM: grupo de *twin block* reverso fijo modificado, OJ; *overjet*, OB: *overbite*, GPM: grupo de un aparato de protracción maxilar

### Proceso de calibración

El proceso de calibración fue realizado por la investigadora principal (KMS) y la segunda autora (GRGT). Para ello, se seleccionaron 10 estudios y se obtuvo un 100% de concordancia, tanto en la búsqueda bibliográfica como en la extracción de los resultados de interés.

## RESULTADOS

Identificación de los estudios: Se identificaron 146 artículos en las bases de datos PubMed (n=21), SCOPUS (n=104) Science Direct (n=7) y Embase (n=14) hasta el 13 de diciembre del 2022. Se eliminaron 13 artículos duplicados con el gestor de citas RefWorks obteniéndose 133 artículos, de los cuales 116 estudios fueron eliminados porque eran revisiones (n=3), reporte de casos (n=4), no respondían a la pregunta de interés (n=102), comentarios (n=2), estudio en animales (n=3) y estudio en cráneo (n=1), obteniéndose 17 artículos, de los cuales fueron excluidos por no cumplir con los criterios de selección (n=2) y en otro idioma (n=3). Finalmente se seleccionó de 12 artículos para el análisis. (Figura 1)

Características demográficas y clínicas de la muestra: Casi todos los estudios detallaron la condición de la muestra clase III analizada según el ángulo ANB menor a 0° 9,24 o menor a -1° ^8^ y según Witts menor de 1,5 mm, ^9^ menor de -1 mm ^27^ o menor de -2 mm ^24^. La MCA fue evaluada por un over jet negativo (< 0 mm) ^9^^,^^27^ o de -1,7 mm, ^25^ mordida borde a borde ^8^^,^^24^ y consideraron el overjet negativo que comprometieran tres a cuatro dientes antero inferiores sobre los dientes antero superiores. ^2^^,^^8^^,^^22^^,^^23^^,^^24^ Algunos estudios incluyeron el prognatismo mandibular, ^(19.24)^ retrognatismo maxilar, ^8^^,^^22^^,^^23^ patrón de crecimiento horizontal ^8^^,^^9^ y relación molar Clase III según Angle ^9^^,^^19^^,^^27^ o Superclase I ^9^.

### Efectos de ATM con aparato de protracción maxilar

Cuatro estudios evaluaron dos diferentes aparatos de protracción maxilar (de Grummons y de Delaire). La cantidad de pacientes estudiados fue variada y oscilaba de 17 a 35 pacientes con máscara facial de Delaire (MFD), 16 pacientes con máscara facial de Grummons (MFG) y el grupo control (GC), con 13 a 38 pacientes. La fuerza utilizada fue diferente en cada aparato; en la MFD se utilizó 400 g/lado, con un vector de fuerza de 20°-35° ^(8, 22, 23)^, 300-350 g/lado en la MFG con un vector de fuerza de 20°-25° ^9^. El tiempo de uso de los dos tipos de aparatos comprendía de 14-16 h/día. Los resultados cuantitativos de la posición del cóndilo entre la MFG y la MFD mostraron disminución para posición anteroposterior del cóndilo (∆X) y posición superoinferior del cóndilo (∆Z) del lado izquierdo y derecho, después de 8 a 10 meses de tratamiento (p < 0,01), así como hubo diferencias significativas en ∆X del lado derecho e izquierdo, y ∆Z del lado izquierdo, evaluados de máxima intercuspidación (MI) a relación céntrica (RC) (p = 0,046; 0,044 y 0,007 respectivamente) ^9^. En otro estudio comparativo entre MFD y GC se demostró un aumento ligero de clic (del 5,7% al 9,1%) en MFD y el dolor lateral en el ATM y espasmo pterigoideo lateral fue ≤8,6% en ambos grupos y tiempos después de 15 meses de tratamiento; pero se halló una disminución (≤3%) del dolor intraarticular, bloqueo y pérdida de movimiento, o espasmo masetero/temporal ^22^. Por otro lado, se evidenció una disminución de la prevalencia de DTM del 2% al 0%, y de la crepitación en un 9,3% al comparar ambos tiempos y grupos (MFD y GC) después de 3 años, según Mandall *et al*. ^23^.

### Efectos de ATM con mentonera

Seis estudios compararon la mentonera (M) con un grupo control (GC) de oclusión normal ^25^ y/o clase III esqueletal ^18^^,^^24^^-^^26^. La cantidad de pacientes con M estudiados oscilaba de 19 a 86 pacientes, mientras que el GC comprendió de 16 a 53 pacientes. La fuerza aplicada varió de acuerdo con el estudio: 200-250 g/lado ^18^, 300 g/lado ^24^, 500 g ^25^, 400-500 g ^17^ y 600 g/lado ^27^ de tracción occipital. La mayoría de los estudios demostró que el tiempo de uso de la mentonera oscilaba de 7 a 24 h/día ^17^^,^^18^^,^^24^^,^^25^^,^^27^ y uso nocturno como tratamiento de contención ^18^. Los resultados cuantitativos entre la mentonera y GC demostraron que los pacientes tratados con mentonera presentaron un movimiento vertical del punto condylion (-0,1 mm/año) a diferencia del GC de -0,5 mm/año (p > 0,05), después de 28 ± 7,9 meses ^18^. En los resultados cualitativos, se demostró la compresión anteroposterior de la distancia relativa entre los cóndilos y proceso coronoides después del uso de la M que en el GC ^24^. La presencia de dolor de ATM fue mayor en el GC con oclusión normal (54,5%) que en GM (37,5%) (p < 0,01) después de 2 a 11 años de tratamiento ^25^. El estudio de Deguchi *et al* ^.(^^17^ demostró ausencia de DTM en un 67,4% (58 de 86), dolor espontáneo en ATM en un 3% aproximadamente, clic en la ATM en un 9% aproximadamente, y se no observaron sonidos articulares o alteración en la apertura de los pacientes después de ser tratados con mentonera. Asimismo, el estudio de Wendl *et al* ^.(^^27^ no reveló signo o síntoma de DTM. Por otra parte, se observó cambios en el ángulo de la cabeza del cóndilo, altura del cóndilo, altura de fosa (p < 0,05), el ancho de la fosa y espacio anterior del cóndilo (p < 0,01) cuando se compararon ambos tiempos, y después del tratamiento, al comparar el GC y M, se halló cambios en el ángulo de la cabeza del cóndilo, altura de la fosa, ancho de la fosa, espacio anterior del cóndilo y altura del cóndilo (p < 0,05) ^26^.

### Efectos de ATM con aparatos funcionales

Tres estudios analizaron la presencia de cambios en el ATM, el aparato funcional evaluado fue el *twin block* reverso (TBR) ^2^^,^^19^ y el *jasper jumper* modificado (JJM) ^8^. La cantidad de pacientes en el TBR estudiado oscilaba de 20 a 32. El tiempo de uso fue de 22 h/día, excepto durante las comidas, para TBR ^19^ y 14 h/día para el JJM ^8^. Los resultados cualitativos revelaron un incremento de la distancia entre espacios articulares en el TBR a diferencia del TBR más terapia de fotobiomodulación (TF) (p < 0,05), un aumento del volumen condilar en TBR (p<0,01) y ambos grupos (TBR y TBR más TF) presentaron una disminución del espacio articular posterosuperior y un aumento del espacio articular anterior y mediolateral (p < 0,001) después de 258,68 ± 16,43 días de tratamiento ^19^. En el estudio de Liu *et al*. ^2^ se demostró que los pacientes diagnosticados con DTM se aliviaron después de un mes de tratamiento y en 4-5 meses de uso del TBR fijo desaparecieron el chasquido articular, el dolor y la cefalea en 18 pacientes diagnosticados con DTM, y esto se mantuvo después de 3 años. De igual modo, se demostró una disminución del dolor muscular y de la ATM al comparar el pre y postratamiento del *jasper jumper* modificado (JJM) luego de 4 meses de tratamiento (p < 0,01 y p < 0,05) ^8^.

### Comparación de los efectos de ATM entre los aparatos ortopédicos

Después del tratamiento, se demostró una baja prevalencia de dolor en la ATM después del uso de la máscara facial de Delaire ^22^^,^^23^, del JJM ^8^, de la mentonera ^17^^,^^25^^,^^27^ y del *twin block* reverso fijo ^2^. También se evidenciaron signos y síntomas de DTM después del tratamiento en menor porcentaje, como el clic, del 5,7 % al 9% con la MFD ^22^ y del 9% con la mentonera ^17^; crepitación en un 15,8%, dolor lateral en la ATM y espasmo pterigoideo ≤ 8,6% en MFD ^22^. Del mismo modo, se observó dolor en la ATM en un 3% y ausencia de sonidos articulares y alteración en la apertura después del tratamiento con la mentonera. Además, hubo cambios en la morfología del cóndilo en pacientes tratados con mentonera ^(26, 27)^, en la posición del cóndilo en MFD y MFG ^9^. De igual manera, hubo cambios en el espacio articular en pacientes tratados con *twin block* reverso ^19^.

## DISCUSIÓN

El propósito de este estudio es dar a conocer los efectos que se producen en la ATM después del tratamiento ortopédico en pacientes clase III con mordida cruzada anterior, por medio de una revisión exhaustiva de artículos que cumplían con los criterios de selección preestablecidos. Sin embargo, se encontró artículos que no presentaban una información detallada y escasos estudios sobre el tema.

Los estudios analizados en el presente trabajo de investigación demostraron cambios significativos en la posición del cóndilo después del tratamiento con aparatos de protracción maxilar, lo que también se presentó en el estudio de Lee *et al* ^.(^^14^, los cuales fueron la remodelación de la cavidad glenoidea, la aposición de la pared frontal y la reabsorción de la pared posterior, que llevó al cambio en la posición del cóndilo después del tratamiento con la MFD. De los estudios evaluados, hubo una baja prevalencia de dolor en la ATM y dolor muscular después del uso de la máscara facial de Delaire de 400 g/lado, con un vector de fuerza de 20°-30°, durante 6 meses a un año y 3 meses de tratamiento, con o sin signos y síntomas previos en la ATM. Debemos reconocer que la magnitud y la dirección de la fuerza son importantes tanto para un crecimiento maxilar eficiente como para el desplazamiento anterior de este ^13^^-^^16^; pero no existen estudios que determinen la fuerza óptima para el tratamiento con aparato de protracción maxilar y que, a su vez, no produzcan DTM, como lo mencionan en otras investigaciones ^13^^,^^31^.

En esta revisión se encontró baja prevalencia de DTM, pero cambios en el ángulo de la cabeza del cóndilo, la altura de cóndilo y la altura de fosa después del tratamiento con la mentonera, al igual que lo demostraron Gokalp y Kurt después de ocho años nueve meses de tratamiento con mentonera ^28^ y Gokalp *et al*. después de dieciséis meses de tratamiento ^29^. Además, encontramos que cada autor aplicó diferentes fuerzas en el protocolo de tratamiento con mentonera y que, a pesar de que se aplicaron fuerzas mayores a 400 g, estas no evidenciaron dolor de la ATM después del tratamiento, como se concluyó en otros estudios ^28^^-^^30^.

El *twin block* reverso (TBR) ha demostrado ser una alternativa efectiva para la corrección de la mordida cruzada anterior. Según Khwanda *et al*. ^19^, el *twin block* reverso puede producir un aumento de volumen condilar, una disminución del espacio articular posterosuperior y el aumento del espacio articular anterior y mediolateral, con o sin el uso del tratamiento de fotobiomodulación, el cual es un método de aceleración del movimiento ortodóntico. El estudio de Liu *et al*. ^2^ demostró la ausencia de dolor en la ATM en pacientes diagnosticados con DTM después de 4-5 meses de tratamiento con TBR fijo y ausencia de DTM después de 3 años del periodo de seguimiento. El *jasper jumper* modificado, otro aparato funcional, ha demostrado también disminución de dolor muscular y de ATM al comparar del pre y postratamiento, usando una fuerza de 200 g/lado, 14h/día por 4 meses. No se ha podido encontrar más evidencia sobre el efecto del TBR y JJM en la ATM.

## CONCLUSIONES

No existe evidencia científica suficiente que determine la ausencia o presencia de signos y síntomas, así como cambios en la ATM, después del tratamiento con máscara facial de Grummons y de Delaire, aparatos funcionales como el *twin block* reverso y el *jasper jumper* modificado, además del seguimiento postratamiento de los aparatos ortopédicos en pacientes con maloclusión clase III con mordida cruzada anterior.

## Fuente de financiamiento:

El presente trabajo de investigación fue autofinanciado
